# Botanical Description of British Plants

**Published:** 1805-07-01

**Authors:** 


### *Botanical Description of British Plants.*

( Continued from vol. xii. pp. 554—560. )

PENTANDRIA. PENTAGYNIA.

69. LINUS. *L. usitatissimum. L. arvense. L. sativum. Ang.* Flax.

*Gen. Desc.* Cal. five leaved. Petals five. Caps. five-valved, ten-celled. Seeds solitary.

*Spec. Desc.* *Leaves* alternate, spear-shaped. *Cal. caps.* dagger-pointed. *Petals* scolloped, sky blue, scored with darker lines. *Stem* generally solitary, cylindrical. *Filam.* united at base. Styles blue, thicker towards the top. Inner edge of cup fringed. *Corn fields, sandy pastures.* BLOSS. July.

*Use.* This valuable plant came originally from those parts of Egypt that are exposed to the inundations of the Nile; and it is said there to rise with a strong stem to the height of four feet: but it is now a native of Britain, and its utility in arts, manufactures, and medicine, have rendered the cultivation of it an object of particular encouragement. The *seeds* have an unctuous mucilaginous smell, and yield, by expression only, a large quantity of oil, which when carefully drawn, without the application of heat, has no particular taste or flavour, and is an excellent pectoral. Boiled in water, they afford a large proportion of strong flavourless mucilage: infusions and decoctions of them, like other vegetable mucilages, are used as *emollients* or *demulcents* in hoarsenesses, coughs, and pleuretic symptoms, which frequently prevail in catarrhal affections: they are also recommended in nephritic pains and stranguaries: for these purposes a spoonful of the seeds unbruised is said to be sufficient for a quart of water. The seeds are also much used externally in *emollient* and *maturating cataplasms*, and make an easy and useful poultice in cases of external inflammation. The expressed oil is supposed to be of a more healing and balsamic nature than other oils of this class; and has therefore been very generally employed in pulmonary complaints, also in cholics, and constipations of the bowels: in some of its prp erties it differs considerably from mo t other oils of this kind; not congealing in winter, not forming a solid soap with fixed alkaline salts; and acting more powerfully than any other as a menstruum on sulphureous bodies. When heat is applied during the expression, it gets a yellowish colour and a peculiar smell: and in this state it is used by painters and varnishers. Linseed appears to afford but little nourishment, and when taken as food has been found to impair the stomach and produce flatulency: these effects are noticed by Galen, and have since been confirmed by the experience of Tragus, during a season of scarcity in Zealand, where boiled linseed was used as food, and occasioned remarkable distention of the hypochondria, swellings of the face, and other parts, which in some instances proved fatal. *Ray, Hist. p.* 1073. But the farinaceous part which remains after the oil is expressed, called *oil-cake*, is given to oxen with advantage, and they soon fatten upon it. The longitudinal fibres of the bark, separated by maceration in water from the glutinous matter which connects them together, dried, beaten, and combed, are spun and manufactured into linen: of this linen, when worn to rags, is made paper. The water in which flax has been macerated is poisonous to cattle, and therefore the practice of steeping it in any running stream or common pond is prohibited by statute. See 33 Hen. viii. chap. 17. —*Woodville. Withering.*

70. Linus. *L. catharticus.*

*Ang.* Purging flax. Mill mountain. Dwarf wild flax.

*Gen. Desc. As above.*

*Spec. Desc. Leaves* opposite, egg-spear shaped. *Stem* forked. *Bloss.* pointed. Before the flowers open they hang down. *Cal.* edge fringed with minute glands on foot stalks. *Filam.* united in a bag inclosing lower half of germen. *Dry pastures.* Bloss. May, July.

*Use.* A drachm of the dried plant pulverised, or an infusion of a handful of it in whey or water, (*Lightfoot*) or an infusion of two drachms or more of the dried plant, is an excellent and safe *purge*, and has been given with advantage in obstinate *rheumatisms.* It frequently acts as a *diuretic.*—*Withering.* A decoction of the whole plant is a very rough purge, much used among the country people. —*Hill.* Horses, sheep, and goats eat it.

71. Drosera.

71. Drosera. *D. rotundifolia. Ros solis.*

*Ang.* Round leaved sundew. Red rot. Youth wort. Moor grass.

*Gen. Desc.* Cal. five div. Pet. five. Caps. one cell, five valves at top. Seeds several, fixed to the sides.

*Spec. Desc. Stalks* from the root. *Leaves* circular. *Styles* six. *Leaf-stalks* fringed at the base. *Blossom,* white. *Mossy bogs.* Bloss. July, Aug.

*Use.* The whole plant is acrid, and sufficiently caustic to erode the skin; but the ladies know how to mix the juice with milk, so as make it an innocent and safe application to remove freckles and sun-burn. The juice that exudes will, unmixed, destroy warts and corns: this plant has the same effect upon milk as the pinguicala vulgaris; and like that too, is supposed (particularly in the Scotch Highlands, *Lightfoot)* to occasion the rot in sheep. The name of *sundew* seems to be derived from the striking appearance of these plants; the leaves being fringed with hairs supporting small globules of pellucid liquor like dew, which continue even in the hottest sun. Perhaps its acrimony resides in this secreted liquor.—*Withering.*

### Class VI. Hexandria.

### Hexandria. Monogynia.

1. Allium. *A. arenarium.*

*Ang.* Sand garlic.

*Gen. Desc.* Bloss. six pet. expanding. Sheath dry, skin-like, many flowered; umbel, crowded; caps, superior, three-celled.

*Spec. Desc. Stem-leaves flat, umbels bearing bulbs. Umbel-sheath,* pointless. *Stamens,* three-pointed; *half-sheaths,* cylindrical. *Bulbs and blossoms,* blue or purple. *Stem,* two to four feet high. *Mountains in Westmoreland.* Bl. June, July.

*Use.* This is eaten along with other pot herbs. It communicates its flavor to milk and butter of cows that eat it. —*Withering.*

2. Allium. *A. oleraceum.*

*Ang.* Wild garlic.

*Gen. Desc. As above.*

*Spec. Desc. Leaves semi-cylindrical, hollow; umbels bearing bulbs.* Filamens undivided. *Root* a solid bulb, egg-shaped, numerous. *Stem* two or three feet high, bent at the top, smooth, solid. *Bloss.* pale green with purple lines. *Pastures, Cornfields.* Bl. July.

*Use.*

*Use.* The tender leaves are very commonly boiled in soups, or are fried with other herbs. Cows, goats, sheep and swine eat it.—*Withering.*

3. Allium. *A. ursinum.*

*Ang.* Ramsons. Ramson garlic.

*Gen. Desc.* *As above.*

*Spec. Desc.* *Leaves from the root*; spear-shaped on leaf-stalks. *Stalk naked;* three-square, below cylindrical. *Umbel* flat-topped. *Flowers* large, numerous, white. *Woods, hedges, dry meadows.* Bloss. May.

*Use.* An infusion of this plant in brandy is esteemed a good remedy for the gravel. *Pennant,* 1772, *p.* 175. Other plants do not flourish in its vicinity.—Cows eat it, but it communicates its flavour to their milk and butter, so strongly as to be very offensive in the spring.—*Withering.*

*Note.* The garlic commonly cultivated in our gardens, which is a native of Sicily, (A. sativum) is that which is much used in medicine: it is an expectorant in pituitous asthmas, &c.; as a diuretic in dropsies; as a febrifuge in preventing the paroxsysms of intermittents, and even subduing the plague: as an anthelmintic, an antiscorbutic, and as a powerful lithontriptic: it has also been applied externally to tumours and cutaneous diseases, and has been used successfully in certain cases of deafness. *Woodville. See Hoffman, Rosenstein, Tauke. Lind, Lobb de dissol. calc.* c. 10. *Sydenham de variolis conf. Bergius, and others.*

4. Ornithogalum. *O. luteum. Bulbus Sylvestris.*

*Ang.* Yellow Bethlem Star. Star of Bethlehem.

*Gen. Desc.* Bloss. six petals, upright, permanent, above the middle expanding; filaments, alternately dilated at the base; caps, three-celled.

*Spec. Dec.* *Stalk* angular, two-leaved, four to six inches high. *Fruit-stalks* in an unbranched umbel, sometimes solitary. *Root-leaf* generally single, longer than the stem: stem-leaves three or four, unequal. *Leaves* fringed with fine hairs. *Umbel-sp.* three to seven, each with one flower. *Petals* in two series, the inner greenish yellow, the outer green. *Moist sandy places; wet woods.* Bl. April.

*Use.* The bulbous roots of this whole genus are nutritious and wholesome, and those of this species have been employed for food in times of scarcity of provisions.—Horses, sheep and goats eat it: swine are not fond of it: cows refuse it. *Withering.*

5. Narthesium. N. *ossifragum.* *Asphodelus Lancastriæ.*

*Ang.* Lancashire asphodel. Bastard asphodel. Lancashire king's spear.

*Gen. Desc.* Bloss. six pet. permanent. Style 0. Caps. egg-shaped. Seeds tapering at each end.

*Spec Desc.* *Stem* ascending, four to nine inches high. *Pet.* strap-spear-shaped, concave, expanding, outside greenish, yellow within. *Filam.* woolly, yellow. *Anthers* scarlet. *On turf bogs.* Bl. July, August.

*Use.* This plant is believed in Sweden to be noxious to sheep: and is supposed to soften the bones of animals that eat it; but this opinion wants confirmation. Cows and horses eat it. Sheep and swine refuse it.—*Withering.*

6. Asparagus. *A. officinalis.*

*Ang.* Asparagus, Sparagus, Sparrowgrass, Sperage.

*Gen. Desc.* Bloss. six div. upright. Inner petals, tops reflected. Berry sup. three-celled. Seeds two.

*Spec. Desc.* *Stem* herbaceous, cylindrical, upright, panelled. *Leaves* bristle-shaped; leaf-scales solitary or in pairs. M. and F. fl. sometimes on distinct plants, but not when cultivated. *Bloss.* bell-shaped, yellow green; fl. M. Fem. or hermaph. *berries* red: seeds one to three. *Meadows and rocks on the sea coast.* Bl. July.

*Use.* The young shoots, in a cultivated state, are universally esteemed for their flavor and nutritious qualities. They impart to the urine the scent that is perceived on the water in which they have been boiled.—*Withering.*

7. Convallaria. *C. majalis.*

*Ang.* Lily of the valley. May Lily. Lily Solomon's Seal.

*Gen. Desc.* Bloss. six-cleft. berry three-celled, superior. Seeds two.

*Spec. Desc.* *Bloss. bell-shaped,* contracted at the mouth, segments reflected, white, highly fragrant. *Stalk* naked, semi cylindrical; *flowers* spiked, nodding, few, growing from one side. *Berries* red. *Woods, heaths.* Bl. May.

*Use.* The flowers, though highly fragrant, have, when dried, a narcotic scent. Reduced to powder they excite sneezing. An extract prepared from the flowers, or from the roots, partakes of the bitterness, as well as of the *purgative* properties of aloes: the dose from twenty to thirty grains.—By the assistance of lime a beautiful and durable green colour may be prepared from the leaves.—Sheep and goats eat it: horses, cows and swine refuse it.—*Withering.*

8. Convallaria. *C. polygonatum.*

*Ang.* Sweet smelling Solomon's Seal.

*Gen.*

*Gen. Desc.* *As above.*

*Spec. Desc.* *Leaves* alternate, embracing the stem. *Stem* two-edged. *Fruit-stalks* axillary, mostly one-flowered, long, hanging on the opposite side. *Flowers* white, with a green line on each segment, bent in. *Berries* black. *Rocky woods.* Bl. May, June.

*Use.* The root is used in Medicine: it is of a mucilaginous quality, and has long been used as a discutient poultice to various kinds of tumours, but more particularly to bruises accompanied with extravasated blood in the cellular membrane. It is also recommended as a cosmetic; and in Galen's time, was used by women to remove pimples and freckles from the skin. Of its astringent effects internally, there can be no well-grounded expectation. The berries, flowers and leaves are extremely acrid, and are said to be of a poisonous quality.—*Woodville.* In seasons of scarcity the roots, which contain a considerable proportion of farinaceous matter, have been used for making bread.—*Bergius, Withering.* Horses, cows and swine refuse it.

9. Convallaria. *C. multiflora.*

*Ang.* Common Solomon's Seal.

*Gen. Desc.* *As above.*

*Spec. Desc.* Leaves alternate, embracing a cylindrical stem. Fruit-stalks axillary, many-flowered, branched. Flowers smaller than C. Pol. white tipped, with green. Berries black. *Woods.* Bloss. May, June.

*Use.* The roots of this species also have been employed for making bread; and its young shoots are eaten by the Turks as Asparagus.—*Withering.* To this species the root used in medicine is very generally referred by writers on the Materia Medica.—*Woodville.*

10. Hyacinthus. *H. non-scriptus.*

*Ang.* Harebell hyacinth. English hyacinth.

*Gen. Desc.* Bloss. bell-shaped, permanent; segm. rolled back: germen with three nectariferous pores at the top.

*Spec. Desc.* Bloss. tubular-bell-shaped, six deep divisions, pendent, fine blue, sometimes white: *floral-leaves* in pairs. *Woods, hedges.* Bl. May.

*Use.* The roots of this plant serve well for the manufacture of starch. When fresh they are poisonous.—*Withering.*

11. Acorus. *A. calamus.*

*Ang.* Myrtle flag. Sweet smelling flag, or calamus. Sweet myrtle grass.

*Gen. Desc.* Spadis cylindrical, covered with florets; pet. six, naked. Style 0. Caps. three celled.

*Spec. Desc. Floral-leaf* much longer than the spike. *Sp.* three inches long, closely studded with florets set spirally. *Root* decays annually, two new ones shooting from the crown. *Stem* flatted, bordered with a leafy edge, above the insertion of the spike expanding into a leaf. *Leaves* sword-shaped, sheathing each other, some plaited in a serpentine line. *Spike* proceeding from the edge of the leaf. *Petals* skinny, thin, crowned with a kind of horizontal hat. *Banks of rivers with a muddy bottom, stagnant waters.* Bl. May, June.

*Use.* The root, in its dried state, has a moderately strong aromatic smell, and a warm pungent bitterish taste. Water is found to extract the bitter, and rectified spirit the aromatic matter. Though not heating, like spices, this root, manifesting to the taste considerable pungency and a moderate share of bitterness, has been therefore deemed useful as a warm stomachic, and was formerly much used here in combination with the more simple bitters, which, by this addition, were rendered more grateful and carminative. It has been recommended in vertigo proceeding from a vitiated stomach, and in intermittent fevers, which are said to have been cured by this root after the bark had failed. It is also said to be efficacious in scorbutic and hæmorrhagic complaints; but to this little credit will be given, and still less to its supposed alexipharmic power.—*Woodville.* This plant grows wild in moist places in Japan, where it was considered, on account of its strong aromatic taste, to be a medicine of great powers; but they do not there know its true and proper use.—*Mr. Hincks.* It is the only truly aromatic plant which is a native of northern climates.—*Linn.* The root powdered might supply the place of foreign spices; it has cured agues, when the Peruvian bark has failed: the flavor of the root is greatly improved by drying. This root is commonly imported from the Levant, but those of our own growth are full as good. The Turks candy the roots, and think they are a preservative against contagion. Neither horses, cows, sheep, goats, nor swine will eat it.—*Withering.*

12. Tamus. *T. communis.*

*Ang.* Bryony Lady-seal. Black briony.

*Gen. Desc.* Fl. m. and fem. in different plants. Cal. six-div. Bloss. 0. Fem. style three-cleft. berry three-celled, beneath. Seeds two.

*Spec. Desc. Root* large. *Stem* twining. *Bloss.* greenish; *berry*

*berry* red. *Thickets.* Bl. June. The leaves vary from kidney to heart, heart-spear, triangular-spear, or halberd-shaped.

*Use.* The root is acrid and stimulating. The young shoots are a good eatable, when dressed like asparagus. Horses will not eat it.—*Withering.*

13. Berberis. *B. vulgaris.* *B. dumetorum.*

*Ang.* Barberry. Pipperidge Bush.

*Gen. Desc.* Cal. six-leaved. *Petals* six, two glands to the claw. Style 0; berry superior, one-celled, open at the end. Seeds two or three.

*Spec. Desc.* *Fruit-stalks* forming bunches: thorns three together. *Leaves* change into three-forked thorns, and are succeeded by fresh leaves the next year: anthers burst at the least touch, or at any irritation to the bottom of the filament bend over to the summit. *Bloss.* yellow, streaked with orange. *Berries* red. *In woods and hedges, on chalk hills.* Bl. May, June.

*Use.* The berries, which are gratefully acid and moderately restringent, are said to be of great use in *bilious fluxes,* and in all cases where heat, acrimony, and putridity of the humours prevail. The Egyptians are said to employ them in *pestilential fevers* and *fluxes* with great success. (*Alpinus Med. Ægypt. L.* 4. *c.* 1.) S. Paulli relates that he was cured of a malignant fever, accompanied with a bilious diarrhœa, by using these berries conformably to the Egyptian practice, viz. macerating the fruit for a day and a night in twelve times its quantity of water, with the addition of a little fennel seed; the liquor strained and sweetened, was used as a common drink. (*Inadrip. Bot.* 118.) These berries do not seem, however, to possess any peculiar advantage over other acid fruits.—*Woodville.* The inner bark steeped in white wine is purgative, and has often been found serviceable in the jaundice. The fruit is cooling and good to quench thirst in fevers, for which it is generally made into a conserve.—*Lightfoot.* An infusion of the bark in white wine is purgative. The leaves are gratefully acid. The flowers, though offensive to the smell when near, afford, at a proper distance, an odour extremely fine. The berries are so acid that birds will not eat them, but boiled with sugar, they form a most agreeable rob or jelly; they are likewise used as a dry sweetmeat and in sugar plumbs. The roots, boiled in lye, dye wool yellow: in Poland, with the bark of the root, leather is dyed of a most beautiful yellow: the inner bark of the stems, with the assistance of allum, dyes linen a fine

 yellow.

yellow.—This shrub should never be permitted to grow in corn lands, for the ears of wheat, that grow near it, never fill, and its influence in this respect has been known to extend as far as three hundred or four hundred yards across the field. Of this, though denied by Mr. Brousonet, *see Eng. Bot. p.* 49, Dr. Withering has experienced the truth.—*Withering.*

Hexandria Trigynia.

14. Rumex. *R. crispus.*

*Ang.* Curled Dock.

*Gen. Desc.* Calyx three-leaved. Petals three, closing. Seed one, three-cornered, inclosed in the blossom.

*Spec. Desc. Fl. herm. Pet. entire, all beaded. Valves* strongly veined. *Leaves* spear-shaped, acute, waved and curled at the edge. *Root* yellow. *Beads* one or three, rarely two. Valves large brown red when ripe. Beads pale when young, changing to blood red, then to brown red. *Pastures, road sides, all soils.* Bl. June, July.

*Use.* The fresh roots bruised and made into an ointment, or a decoction of them, is a cure for the *itch.* The seeds have been given with advantage in *dysentery.* In Norfolk this plant is the pest of the clover fields. Horses, cows, and goats refuse it.—*Withering.*

15. Rumex. *R. acutus.*

*Ang.*

*Gen. Desc. As above.*

*Spec. Desc. Valves* veinless. *Leaves* oval spear-shaped, uneven at the edges, not entire, not waved or curled. Valves very small, all beaded, entire. Stem and leaves in the sun tinged with red. *Woods, river-sides, roads, commons.* Bl. June, July.

*Use.* The root is much used by dyers; it gives a great variety of shades from straw colour to a fine olive, and a beautiful deep green to cloths which have been previously blued.—*Stokes.* Cows and horses refuse it.—*Linn.*

16. Rumex. *R. hydrolapathum. Lapathum aquaticum.*

*Ang.* Water Dock.

*Gen. Desc. As above.*

*Spec. Desc. Fl. herm. Petals entire, beaded. Leaves* spear-shaped, smooth, acute, very entire, tapering at the base, a little toothed and waved; lower ones to eighteen inches long; *leaf-stalks* semi-cylindrical, lower ones to fifteen inches long. *Stem* five or six feet high. *Root* within white, without black. *Fruit-stalks* encompassed a little below below the middle with an indistinct ring, in half whirls. *Whirls* from alternate sides of stem, surrounded by a skinny sheath. *Cal.* one leaf, three div. *Beads* greenish white or purplish. *Summits* flat, fringed. *Shallow water.* Bl. July, August.

*Use.* The root, which is the part directed for medicinal use, has a strong austere taste, and readily strikes a black colour to a solution of ferrum vitriolatum; it is strongly astringent, and has been much employed both externally and internally for the cure of *scurvy;* especially when the gums are spongy and frequent hæmorrhages supervene; it is recommended also in various other cutaneous defœdations, and in visceral obstructions. The leaves, which manifest considerable acidity, are said to possess a laxative quality, and have been used to obviate costiveness. Many of this genus were formerly officinal herbs, but this species has been esteemed the most efficacious, and is still retained in the Materia Medica by the Edinburgh College, though the great virtues ascribed to it, are now held in doubt.—*Woodville.* This is a medicine of considerable efficacy, both externally applied as a wash for putrid spongy gums, and internally for some species of *scurvy.* In *rheumatic* pains, and chronical diseases owing to *obstructed viscera,* it is said to be useful. The root has some times a reddish tinge, but soon changes to yellowish brown on being exposed to the air.—*Withering.* The powdered root is one of the best things for a *dentifrice* that can be used.—*Murray App. Med. iii.* 344.

17. Rumex. *R. acetosa. Lapathum acetosum. Acetosa pratensis. Oxalis crispa.*

*Ang.* Common sorrell. Sorrell dock.

*Gen. Desc. As above.*

*Spec. Desc. M. and F. fl. on distinct plants. Leaves* oblong, arrow shaped. *Leaf-stalks* purplish. *Bloss.* reddish. *Male cal. leaf* obtuse. *Female cal. leaf* acuminated, reflexed, waved on the margin. *Meadows and pastures.* Bl. June.

*Use.* The leaves have an agreeable acid taste; and have been esteemed *refrigerant, antiscorbutic, and diuretic.* It possesses all the medicinal virtues of oxalis acetoselle, and being easily procured in abundance, may be conveniently substituted for it. Taken in considerable quantity, or used variously prepared as food, sorrel will be found of important advantage where a *refrigerant* or *antiscorbutic* regimen is required; Linnæus says that the Laplanders experience a serum acetosatum to be in this respect an useful and pleasant diet.—*Woodville.* The Laplanders boil the leaves in water, and mix the juice, when cold, in the milk of the rein-deer, which is thus esteemed by them an agreable and wholesome food, and will keep in a cool place for a long while.—*Lightfoot.* The leaves are eaten by us in sauces and sallads. In France they are cultivated for the use of the table, being introduced into soups, ragouts and fricassees. In some parts of Ireland they are plentifully eaten with milk, alternately biting and sipping; the Irish also eat them with fish, and other alcalescent food. The dried root gives out a beautiful red colour when boiled. Horses, cows, sheep, goats, and swine eat it.—*Withering.*

[ To be continued. ]

